# cPKCγ‐mediated down‐regulation of UCHL1 alleviates ischaemic neuronal injuries by decreasing autophagy *via* ERK‐mTOR pathway

**DOI:** 10.1111/jcmm.13275

**Published:** 2017-07-20

**Authors:** Dan Zhang, Song Han, Shizun Wang, Yanlin Luo, Li Zhao, Junfa Li

**Affiliations:** ^1^ Department of Neurobiology and Center of Stroke Beijing Institute for Brain Disorders Capital Medical University Beijing China

**Keywords:** ischaemic stroke, conventional protein kinase C (cPKC)γ, ubiquitin C‐terminal hydrolase L1, autophagy, mammalian target of rapamycin, extracellular signal‐regulated kinase

## Abstract

Stroke is one of the leading causes of death in the world, but its underlying mechanisms remain unclear. Both conventional protein kinase C (cPKC)γ and ubiquitin C‐terminal hydrolase L1 (UCHL1) are neuron‐specific proteins. In the models of 1‐hr middle cerebral artery occlusion (MCAO)/24‐hr reperfusion in mice and 1‐hr oxygen–glucose deprivation (OGD)/24‐hr reoxygenation in cortical neurons, we found that cPKCγ gene knockout remarkably aggravated ischaemic injuries and simultaneously increased the levels of cleaved (Cl)‐caspase‐3 and LC3‐I proteolysis product LC3‐II, and the ratio of TUNEL‐positive cells to total neurons. Moreover, cPKCγ gene knockout could increase UCHL1 protein expression *via* elevating its mRNA level regulated by the nuclear factor κB inhibitor alpha (IκB‐α)/nuclear factor κB (NF‐κB) pathway in cortical neurons. Both inhibitor and shRNA of UCHL1 significantly reduced the ratio of LC3‐II/total LC3, which contributed to neuronal survival after ischaemic stroke, but did not alter the level of Cl‐caspase‐3. In addition, UCHL1 shRNA reversed the effect of cPKCγ on the phosphorylation levels of mTOR and ERK rather than that of AMPK and GSK‐3β. In conclusion, our results suggest that cPKCγ activation alleviates ischaemic injuries of mice and cortical neurons through inhibiting UCHL1 expression, which may negatively regulate autophagy through ERK‐mTOR pathway.

## Introduction

As we all known, ischaemic stroke has high incidence and prevalence with poor outcome 1, 2. Because of a lack of effective therapeutic treatment for ischaemic stroke, much interest is focused on understanding of molecular mechanism underlying endogenous neuroprotection against cerebral ischaemic injury. Recently, cPKCγ in mediating stroke injury has received particular attention. cPKCγ is expressed in the spinal cord and brain as well as exclusively localized in neurons. A noticeable increase in cPKCγ expression is found in the ischaemic penumbra of patients with stroke 3. The cPKCγ membrane translocation (activation) significantly increases under the conditions of MCAO and OGD, suggesting it is involved in a conserved ischaemic response pathway 4, 5. However, the role of cPKCγ in cerebral ischaemic injury has not been defined unequivocally. The activation of cPKCγ is involved in the neuroprotective effects induced by insulin and oestrogen during MCAO ischaemic injury 6, 7. Our previous studies have shown that cPKCγ may participate in hypoxic preconditioning (HPC)‐induced neuroprotection against cerebral ischaemic injury of BALB/c mice 8, 9, 10. However, some studies have reported that cPKCγ activation may exacerbate the cerebral ischaemic injuries. Down‐regulation of cPKCγ membrane translocation by its inhibitor can inhibit the increment in lactate dehydrogenase (LDH) leakage induced by 20‐min. OGD treatment and decrease the decapitation‐induced ischaemic brain injury of C57Bl/6J mice 5, 11. The different effects of cPKCγ may be attributable to the use of different species, models, protocols, measured end‐points and inhibitors. In this study, using cPKCγ gene knockout mice, we tried to further validate the role of cPKCγ in the cerebral ischaemia‐induced injury induced by 1‐hr MCAO/24‐hr reperfusion (R) or 1‐hr OGD/24‐hr reoxygenation (R) treatment.

Our previous study has identified 23 cPKCγ‐interacting proteins in the cortex of HPC‐treated mice using proteomic analysis, including ubiquitin carboxy‐terminal hydrolase L1 (UCHL1) 12. UCHL1, also known as neuronal‐specific protein gene product 9.5 (PGP 9.5), is present in almost all neurons and represents 1–5% of total soluble brain protein 13. It functions as a deubiquitinating enzyme 14, a ubiquitin (Ub) ligase 15 and a mono‐Ub stabilizer 16. Thus, its roles in neuronal cell function/dysfunction have been reported in neurodegenerative diseases, such as Alzheimer's disease (AD), Parkinson's disease (PD) and Huntington's disease (HD) 17, 18, 19, 20. UCHL1 is a small protein (24 kDa) and has a compact and almost globular shape*,* which may facilitate its crossing the blood–brain barrier (BBB) and stability in biofluid allowing for its detection 21. The UCHL1 levels in cerebrospinal fluid (CSF) and serum of rats have been found to significantly increase after 2‐hr or 6‐hr MCAO, but remain normal after haemorrhagic stroke, which indicate that UCHL1 may be a vital biomarker for cerebral ischaemia injury 22, 23. However, how UCHL1 participates in the ischaemic neuronal injuries and whether cPKCγ regulates UCHL1 expression still remain unclear. In this study, the roles of UCHL1 mediated by cPKCγ in the ischaemic neuronal injuries were explored *in vivo* and *in vitro*.

## Materials and methods

### Animals

The C57BL/6J wild‐type (cPKCγ^+/+^) and cPKCγ knockout (cPKCγ^−/−^) mice were purchased from the Jackson Laboratory (Bar Harbor, ME, USA). The experimental procedures were carried out in accordance with the recommendations in the Guide for the Care and Use of Laboratory Animals of the National Institutes of Health and approved by the experimental animal ethics committee of Capital Medical University (Permit Number: 2012‐X‐7). The mice were housed in the home cage under a 12:12‐hr light/dark cycle, with food and water available ad libitum throughout the study.

Experiments were performed at room temperature (20–22°C) on male mice (6–8 W, 18–22 g). All surgeries were performed under sodium pentobarbital anaesthesia, and all efforts were made to minimize suffering. Animals were randomly divided into three groups, namely Naïve group (no treatment), Sham group (no MCAO treatment) and MCAO group (1‐hr MCAO/24‐hr R treatment). The MCAO‐induced ischaemic stroke mouse model was performed as previous reports 24, 25. Briefly, a 5–0 surgical nylon monofilament with its tip (0.22 ± 0.01 mm in diameter) rounded by heat (#1622‐A, Shadong Biotechnology Company, Beijing, China) was gently inserted into the internal carotid artery through the external carotid artery to occlude the middle cerebral artery (MCA, a point approximately 12 mm distal to the carotid bifurcation). After 1‐hr occlusion of the left MCA, the surgical nylon monofilament was carefully removed to restore blood flow and the mice were kept for 24 hrs. In the Sham group, the mice received the same surgical procedures except that the MCA was not occluded. During the surgery, rectal temperature of mice was maintained at approximately 37°C using a heating pad and an overhead lamp.

The cerebral blood flow (CBF) of the MCA supplied area was measured by laser Doppler flowmetry (Perimed Periflux system 5000, Järfälla‐Stockholm, Sweden), whose tip of the probe was fixed to the surface of skull (2 mm posterior to bregma and 6 mm lateral to midline). CBF was expressed as a percentage of the baseline.

According to neurological disability status scale (NDSS), neurological deficit scores were evaluated by an observer blinded to the mouse genotype and treatment at 24 hrs after 1‐hr MCAO. NDSS can be divided into 11 classes from 0 (normal) to 10 (death) 26. The neurological dysfunction of mice was examined at 24 hrs after 1‐hr MCAO. The pole test was performed to assess sensory motor function of the mice, and the time which the mouse takes to descend the ground (T total) was recorded as previously reported 27; the foot‐fault test is a measure of coordination, and the criteria are assessed as the percentage of foot‐fault of the paw to the total number of steps on a wire mesh 28; the cylinder test was performed to examine the unsymmetrical use of the forearms by recording the number of contact for each forelimb, and the ratio for contralateral limb usage is calculated using the formula (L − R)/(L + R + Both) × 100% 29; and the handedness of mice was determined preference for using the paw and the criteria are shown as Laterality Index 30. The mice were trained 3 days before MCAO surgery, meanwhile all training and tests were performed in triplicates each day. The number of mice in each group was 12; half of them were randomly chosen to measure the infarct volume by TTC staining and the others to analyse protein expression using Western blot analysis.

### Measurement of infarct volume by TTC staining

The brains of mice were cut into coronal sections about 1.5 mm thick. The slices were incubated for 15 min. in 2% 2, 3, 5‐triphenyltetrazolium chloride (TTC) (Sigma‐Aldrich, St. Louise, MO 63103, USA) solution at 37°C and then scanned into the computer. The photographs were analysed using Image Pro Plus 6.0 (Media Cybernetics, Silver Spring, MD, USA) according to previous report 31. The cerebral oedema rate is given by the equation: $\text{(S)}=\left(\sum \text{LT}-\sum \text{RT}\right)/\left(\sum \text{LT}+\sum \text{RT}\right)\times 100\%$ where ΣLT and ΣRT represent the volume of left (ischaemic) and right (non‐ischaemic) hemisphere, respectively. Background (B) = volume of TTC unstained white matter in Sham group/total brain volume of Sham group × 100%. To eliminate the interference of oedema rate and background, the infarct rate was evaluated by this equation: $I=\sum \text{SIN}\;\left(1-S\right)/\left(\sum \text{LT}+\sum \text{RT}\right)\left(1-B\right)\times 100\%$, ∑SIN(1−S) represents the total infarct volume after deducting oedema rate.

### Primary cortical neuron culture

The primarily cultured cortical neurons were prepared from postnatal 24‐hr C57BL/6J (cPKCγ^+/+^ and cPKCγ^−/−^) mice. The cortical neurons were dissociated carefully and seeded at a density of 1.2 × 10^5^ cells per well of 6‐well plates in the DMEM medium (Gibco Inc., Grand island, NY, USA) which was replaced by the serum‐free neurobasal medium (Gibco Inc.) with 2% B27 supplement (Gibco Inc.). And the culture medium was subsequently replaced half of fresh medium every 3 days.

The cortical neurons were subjected to 1‐hr OGD/24‐hr R to mimic ischaemic injury *in vitro*. Briefly, the neurons were replaced with glucose‐free DMEM media (Gibco Inc.) and placed in the hypoxia incubator (Thermo Scientific, Marietta, OH, USA) under the condition of 5% CO_2_/2% O_2_/93% N_2_ at 37°C. LDN‐57444 (LDN) (1.0, 5.0, 10.0 or 15.0 μM, S7135, Selleck Chemicals, Houston, TX, USA), MG‐132 (5.0 μM, S2619, Selleck Chemicals) or Bafilomycin A1 (BafA1) (100.0 nM, B1793, MO, USA) was simultaneously added into the medium, respectively.

After 1‐hr OGD exposure, cells were returned to regular neurobasal medium containing 2% B27 under normoxic conditions (5% CO_2_/21% O_2_/74% N_2_) for 24‐hr R. After 24‐hr R, cells were collected for the following experiments. Cell viability was measured using the CellTiter 96^®^ AQueous One Solution Cell Proliferation Assay kit (Promega, #G3580, Madison, WI, USA), and cell death was determined *via* LDH release rate using the CytoTox 96^®^ Non‐Radioactive Cytotoxicity Assay kit (Promega, #G1780) following the manufacturer's instructions.

### Lentiviral transduction

The neurons were transducted with lentiviral vectors containing UCHL1 shRNA gene (LV3‐GFP‐UCHL1 shRNA 1#, 5′‐GAAACTCCTGTGGTACCATCG‐3′; LV3‐GFP‐UCHL1 shRNA 2#, 5′‐GAAGATAGAGCCAAGTGTTTC‐3′; LV3‐GFP‐UCHL1 shRNA 3#, 5′‐GGGTAGATGACAAA GTGAATT‐3′; LV3‐GFP‐UCHL1 shRNA 4#, 5′‐GCTGTCTTCTTGCGTTCTACA‐3′) or the negative control (NC) (LV3‐GFP‐CN, 5′‐TTCTCCGAACGTGTCACGT‐3′) according to the manufacturer's instruction (Sangon Biotech, Shanghai, China) after 3 days of culture. The efficiency of transducing the lentiviral vectors into primary culture of neurons was about 70%. After 72‐hr lentiviral transduction, the neurons were subjected to 1‐hr OGD/24‐hr R treatment.

### Western blot analysis

As previously reported 32, total protein of the tissues from peri‐infarct region or cultured cortical neurons were extracted by lysis buffer. The samples (30 μg) were subjected to 10% or 12% SDS‐PAGE and transferred onto polyvinylidene difluoride membrane (GE Healthcare, Buckinghamshire, UK). The membranes were incubated at 4°C overnight in the solution consisting of primary antibodies against cPKCγ (1:1000, sc‐211, Santa Cruz Biotechnology, Heidelberg, Germany), nuclear factor κB inhibitor alpha (IκB‐α) (1:500, sc‐371, Santa Cruz Biotechnology), phospho‐IκB‐α (Ser 32, 1:250, sc‐7977, Santa Cruz Biotechnology), adenosine 5′‐monophosphate‐activated protein kinase (AMPK) (1:1000, #07‐350, Millipore, St. Louis, MA, USA), extracellular signal‐regulated kinase (ERK) (1:10000, #06‐182, Millipore, St. Louis, MA, USA), UCHL1 (1:1000, #13179, Cell Signaling Technology, Beverly, MA, USA), caspase‐3 (1:1000, #9665, Cell Signaling Technology), LC3 (1:1000, #12741, Cell Signaling Technology), mammalian target of rapamycin (mTOR) (1:400, #2972, Cell Signaling Technology), phospho‐mTOR (Ser2448, 1:250, #2971, Cell Signaling Technology), phospho‐ERK (1:1000, #9101, Cell Signaling Technology), phospho‐AMPK (Thr172, 1:500, #2535, Cell Signaling Technology), glycogen synthase kinase‐3 (GSK‐3)β (1:1000, #9315, Cell Signaling Technology), phospho‐GSK‐3β (Ser9, 1:1000, Cell Signaling Technology), β‐actin (1:10000, #60008‐1‐Ig, Proteintech, Rosemont, IL, USA) and β‐tubulin (1:10000, T2200, Sigma‐Aldrich). Horseradish peroxidase‐conjugated goat anti‐rabbit or antimouse IgG was used as secondary antibodies (1:4000; Jackson Immuno Research, West Grove, PA, USA) for 1 hr at room temperature. Then the SuperSignal^®^ West Pico Chemiluminescent Substrate (#NCI5080, Thermo Fisher Scientific, Waltham, MA, USA) was employed to detect the signals. The amount of proteins were quantified by densitometry and normalized to β‐actin or β‐tubulin, an internal standard.

### Sandwich ELISA

The CSF and serum of mice after 1‐hr MCAO/24‐hr R and the medium of the cultured cortical neurons after 1‐hr OGD/24‐hr R were collected and measured by a commercial enzyme‐linked immunosorbent assay (ELISA) kit (Abnova, #KA3378, Taiwan, China). Experimental procedures were performed according to the manufacturer's protocol. Each sample was measured in duplicate. Optical densities were determined with a microplate reader set to dual wavelengths of 450 nm/540 nm. Standard curves were generated using recombinant UCHL1 proteins in a different dilution series.

### RNA isolation and real‐time quantitative RT‐PCR

Total RNA was isolated from cultured cortical neurons with the RNeasy Lipid Tissue Mini Kit (QIAGEN, #74804, Germantown, MD, USA) according to the manufacturer's instruction. The RNA was used to generate cDNA by Maxima First Strand cDNA Synthesis Kit for real‐time quantitative RT‐PCR (Thermo Fisher Scientific, #K1642) according to the manufacturer's instructions. To detect UCHL1 expression, the 3′‐untranslated region (NM_011670.2, bp 336–545) was amplified with a forward primer: 5′‐CGGCCCAGCATGAAAACTTC‐3′ and a reverse primer: 5′‐GGGACAGCTTCTCCGTTTCA‐3′. Equal amounts of cDNA were used for real‐time quantitative RT‐PCR analysis using the Power SYBR^®^ Green PCR Master Mix (Applied Biosystems, #4367659, Carisbad, CA, USA) according to the manufacturer's instructions. Fluorescent products were detected using a 7500 real‐time PCR thermal cycler (Applied Biosystems). Ywahz (forward primer: 5′‐AGAGTCGTACAAAGACAGCAC‐3′ and reverse primer: 5′‐GAATGAGGCAGACA AAGGTTG‐3′) was used as an internal control 33. Then the relative expression level was calculated using the following equation: relative gene expression = 2^−(ΔCt sample − ΔCt control)^ 34.

### Immunofluorescent staining

The TUNEL staining assay using the *In Situ* Cell Death Detection Kit (Roche, #11684817910, Berlin, Germany) was performed following the manufacturer's protocol. Briefly, the primarily cultured cortical neurons were fixed with 4% paraformaldehyde at room temperature. This was followed by incubating in a permeabilization solution and then incubating with a TUNEL reaction mixture. For the following immunofluorescent staining, fixed cells were incubated with antibody for NeuN to identify neuron (1:500, #ab104224, Abcam, Cambridge, MA, USA) and antibody for glial fibrillary acidic protein to identify astrocyte (GFAP, 1:1000, #Z0334, Dako Denmark A/S, Glostrup, Denmark) at 4°C overnight followed by TUNEL staining. Then cells were exposed to Alexa 594‐conjugated goat anti‐mouse secondary antibody (1:500, Invitrogen, Eugene, OR, USA) and Alexa 350‐conjugated goat anti‐rabbit secondary antibody (1:500, Invitrogen, Eugene, OR, USA) for 1 hr at 37°C in a dark chamber, respectively. The images were captured by a fluorescence microscope, and data were expressed as the ratio of TUNEL‐positive cells to total neurons.

### Statistical analysis

The protein expression levels of cPKCγ and UCHL1 (the band density of target protein/β‐actin or β‐tubulin), the phosphorylation levels of mTOR, ERK, AMPK and GSK‐3β (the band density of phosphorylated form/total protein), the proteolysis levels of caspases (band density of cleaved caspase/total caspase), the ratio of TUNEL‐positive neurons and LC3‐II (band density of LC3‐II/band density of LC3‐I and II) were calculated as 100% in the control group (Naïve *in vivo*/Normoxia *in vitro*), and then the other groups were expressed as percentage of that of control group. Neurological deficits data were analysed using the nonparametric Kruskal–Wallis test. The values were presented as mean ± S.E.M. Statistical analysis was performed using one‐way analysis of variance (anova) followed by all pairwise multiple comparison procedures using Bonferroni test and *P* < 0.05 was considered as statistically significance.

## Results

### cPKCγ could attenuate ischaemic injuries induced by MCAO or OGD treatment

**Figure 1:**
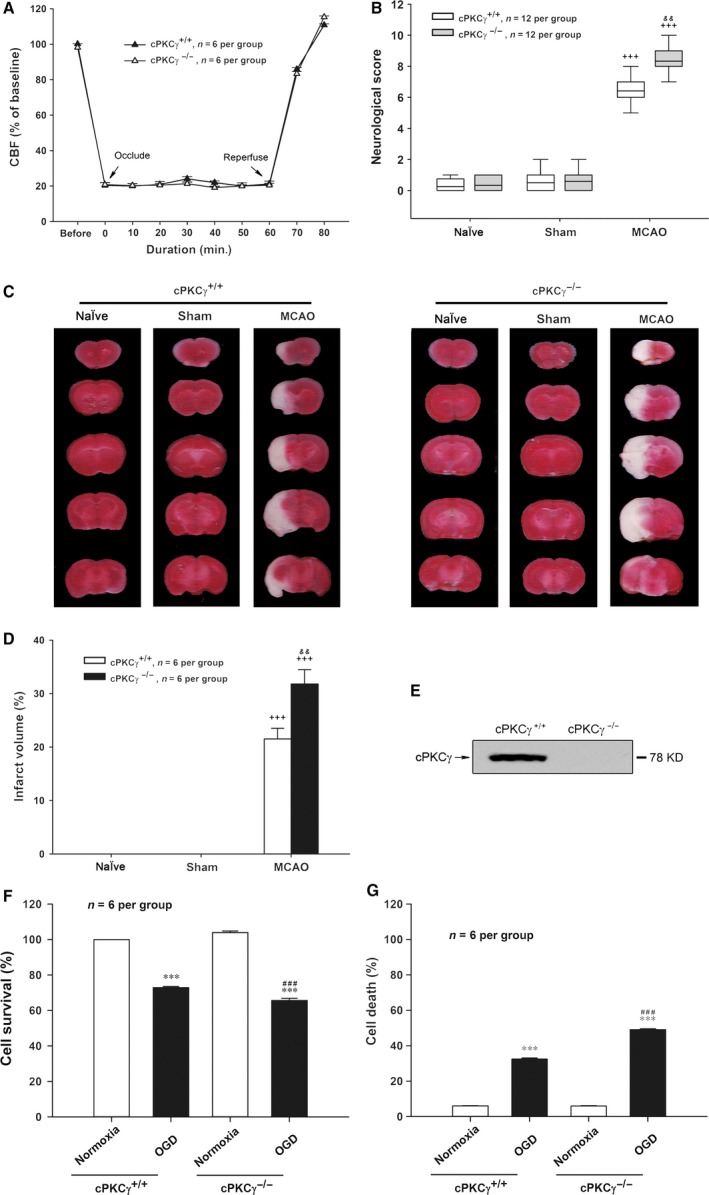
Effects of cPKCγ on regional CBF, neurological score and infarct volume of mice after 1‐hr MCAO/24‐hr R, and cell survival and death rates of cultured cortical neurons after 1‐hr OGD/24‐hr R. (**A**) The change in CBF of mice with MCAO treatment (*n* = 6 per group). (**B**) Statistical results of neurological score (*n* = 12 per group). Representative photographs of TTC‐stained coronal brain sections (**C**) and statistical results of infarct volume (**D**) from Naïve, Sham and MCAO groups of cPKCγ^+/+^ and cPKCγ^−/−^ mice (*n* = 6 per group), respectively. (**E**) Representative results of Western blot demonstrated that cPKCγ was completely knocked out in mice. Quantitative analysis of the cell survival rate (**F**) and the cell death rate (**G**) from Normoxia and OGD groups of cPKCγ^+/+^ and cPKCγ^−/−^ cortical neurons (*n* = 6 per group), respectively. ^+++^ *P <* 0.001 *versus* Sham of cPKCγ^+/+^ mice; ^&&^ *P <* 0.01 *versus* corresponding cPKCγ^+/+^ mice with the same treatment; ***P < 0.001 *versus* Normoxia of cPKCγ^+/+^ neurons; ^###^ *P <* 0.001 *versus* corresponding cPKCγ^+/+^ cortical neurons with the same treatment.

**Figure 2:**
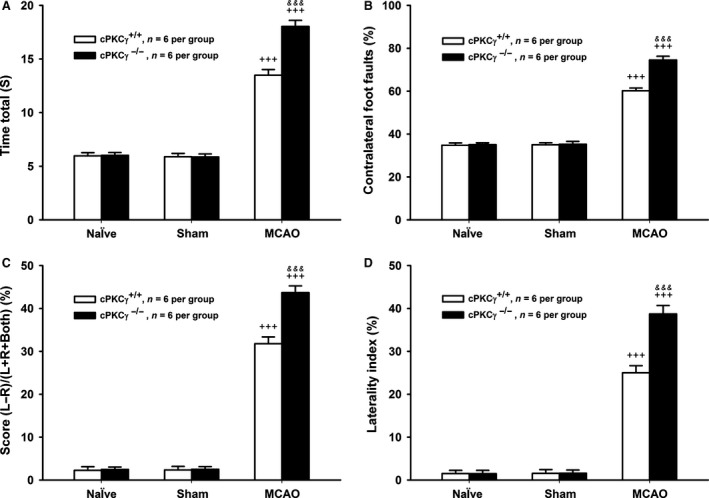
Effects of cPKCγ on the neurological outcome of mice after1‐hr MCAO/24‐hr R. Statistical results of pole test (**A**), foot‐fault test (**B**), cylinder test (**C**) and handedness test (**D**) from Naïve, Sham and MCAO groups of cPKCγ^+/+^ and cPKCγ^−/−^ mice (*n* = 6 per group), respectively. ^+++^ *P <* 0.001 *versus* Sham of cPKCγ^+/+^ mice; ^&&&^ *P <* 0.01 *versus* corresponding cPKCγ^+/+^ mice with the same treatment.

To figure out whether cPKCγ acted as a protective or deleterious molecule during cerebral ischaemic injuries, the regional CBF, neurological deficits and infarct volume were assessed after 1‐hr MCAO/24‐hr R, and the cell survival and death rates were tested after 1‐hr OGD/24‐hr R. cPKCγ was completely knocked out in mice (Fig. 1 E). We found that the regional CBF sharply reduced to about 20% of baseline value after the onset of MCAO and recovered completely after removing the surgical nylon monofilament (Fig. 1A, *n* = 6 per group). There were no significant differences between cPKCγ^+/+^ and cPKCγ^−/−^ mice, indicating that cPKCγ did not affect CBF level during the procedure of 1‐hr MCAO/24‐hr R treatment (Fig. 1A, *n* = 6 per group). We observed that there were no neurological deficits and infarction in the Naïve and Sham groups. The neurological score and infarct volume remarkably increased after 1‐hr MCAO/24‐hr R and further increased in cPKCγ^−/−^ mice compared with that of cPKCγ^+/+^ mice, respectively (Fig. 1B, *P* < 0.001, *P* < 0.01, *n* = 12 per group; Fig. 1C and D, *P* < 0.001, *P* < 0.01, *n* = 6 per group). Meanwhile, results of pole test (Fig. 2A), foot‐fault test (Fig. 2B), cylinder test (Fig. 2C) and handedness test (Fig. 2D) showed that 1‐hr MCAO/24‐hr R remarkably aggravated the neurological deficits of cPKCγ^−/−^ mice compared with that of cPKCγ^+/+^ mice (*P* < 0.001, *n* = 6 per group). In concert with these results of MCAO treatment, 1‐hr OGD/24‐hr R treatment significant decreased the cell survival rate and simultaneously increased the cell death rate compared with that of Normoxia group, respectively (Fig. 1F and G, *P* < 0.001, *n* = 6 per group). Moreover, cPKCγ gene knockout further decreased the cell survival rate and simultaneously increased the cell death rate, respectively (Fig. 1F and G, *P* < 0.001, *n* = 6 per group). These results provide further evidence that cPKCγ may protect neurons against OGD‐ or MCAO‐induced ischaemic injury.

### cPKCγ could decrease the protein expressions of UCHL1 after MCAO or OGD treatment

**Figure 3:**
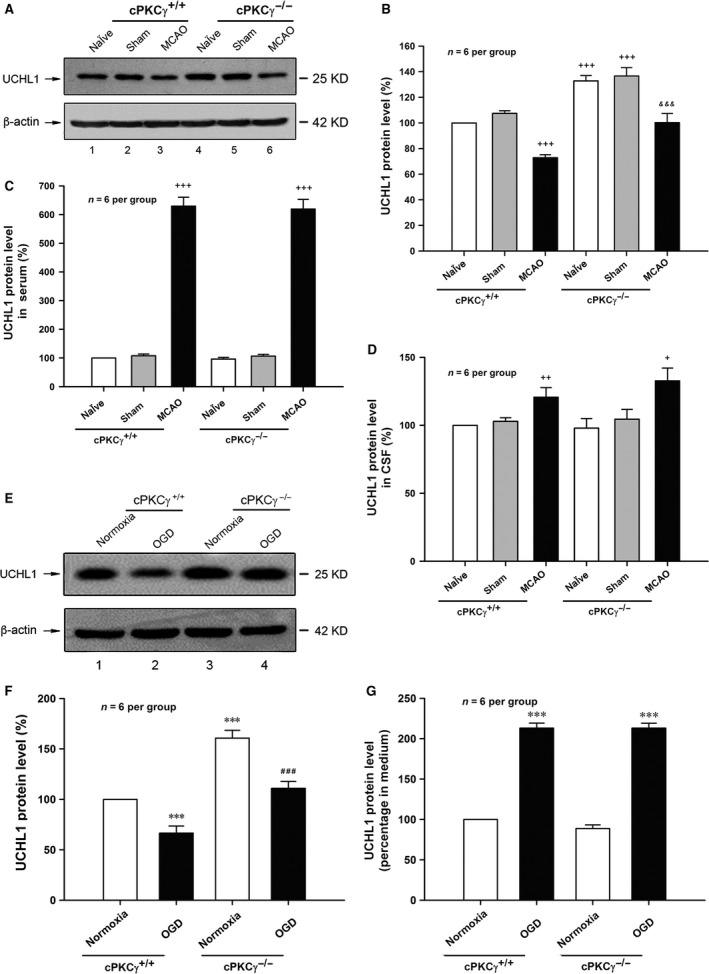
Effects of cPKCγ on the level of UCHL1 after1‐hr MCAO/24‐hr R and 1‐hr OGD/24‐hr R. Representative results (**A**) and quantitative analysis (**B**) of Western blot showed the changes in UCHL1 level in the cortex from Naïve, Sham and MCAO groups of cPKCγ^+/+^ and cPKCγ^−/−^ mice (*n* = 6 per group), respectively. Statistical results of UCHL1 level in serum (**C**) and CSF (**D**) from Naïve, Sham and MCAO groups of cPKCγ^+/+^ and cPKCγ^−/−^ mice (*n* = 6 per group), respectively. Representative results (**E**) and quantitative analysis (**F**) of Western blot showed the changes in UCHL1 level from Normoxia and OGD groups of cPKCγ^+/+^ and cPKCγ^−/−^ cortical neurons (*n* = 6 per group), respectively. (**G**) Statistical results of UCHL1 level in medium from Normoxia and OGD groups of cPKCγ^+/+^ and cPKCγ^−/−^ cortical neurons (*n* = 6 per group), respectively. ^+^ *P <* 0.05, ^++^ *P <* 0.01, ^+++^ *P <* 0.001 *versus* Sham of cPKCγ^+/+^ mice; ^&&&^ *P <* 0.001 *versus* corresponding cPKCγ^+/+^ mice with the same treatment; ***P < 0.001 *versus* Normoxia of cPKCγ^+/+^ neurons; ^###^ *P <* 0.001 *versus* corresponding cPKCγ^+/+^ cortical neurons with the same treatment.

In the next set of experiments, we observed whether cPKCγ could regulate UCHL1 expression or release. Our results showed that the protein expression of UCHL1 obviously decreased, while the levels of UCHL1 in serum and CSF significantly increased after 1‐hr MCAO/24‐hr R (Fig. 3A‐D, *P* < 0.001, *n* = 6 per group). There were no obvious differences in the levels of UCHL1 in serum and CSF between cPKCγ^+/+^ and cPKCγ^−/−^ mice, respectively. However, the UCHL1 protein expression significantly increased in the cPKCγ^−/−^ mice compared with that of cPKCγ^+/+^ mice after 1‐hr MCAO/24‐hr R (Fig. 3A‐D, *P* < 0.001, *n* = 6 per group).

The 1‐hr OGD/24‐hr R treatment also led to increased level of UCHL1 in culture medium and decreased UCHL1 protein expression of primary cortical neurons (Fig. 3E‐G, *P* < 0.001, *n* = 6 per group). cPKCγ gene knockout did not change the level of UCHL1 in culture medium, but significantly increased the UCHL1 protein expression after 1‐hr OGD/24‐hr R treatment (Fig. 3E‐G, *P* < 0.001, *n* = 6 per group). Our results indicate that cPKCγ inhibits UCHL1 protein expression but does not affect the UCHL1 release following MCAO or OGD treatment.

### cPKCγ could decrease the mRNA expression of UCHL1 after OGD treatment

**Figure 4:**
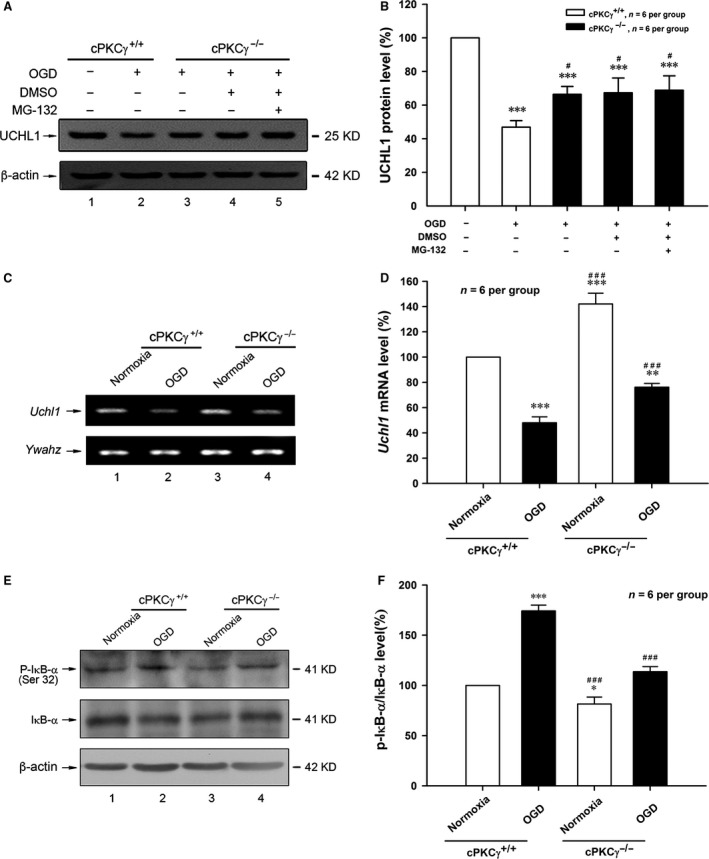
Effects of cPKCγ on the UCHL1 protein and mRNA expression in cortical neurons after 1‐hr OGD/24‐hr R. Representative results (**A**) and quantitative analysis (**B**) of Western blot showed the change in UCHL1 protein expression after MG‐132 (5.0 μM) treatments (*n* = 6 per group). Typical images of PCR (**C**) and quantitative analysis of real‐time quantity RT‐PCR (**D**) showed the mRNA level of UCHL1 from Normoxia and OGD groups of cPKCγ^+/+^ and cPKCγ^−/−^ cortical neurons (*n* = 6 per group), respectively. Representative results (**E**) and statistical results (**F**) of Western blot showed the levels of P‐IκB‐α and total IκB‐α from Normoxia and OGD groups of cPKCγ^+/+^ and cPKCγ^−/−^ cortical neurons (*n* = 6 per group), respectively. *P < 0.05, **P < 0.01, ***P < 0.001 *versus* Normoxia of cPKCγ^+/+^ neurons; ^#^ *P <* 0.05, ^###^ *P <* 0.001 *versus* corresponding cPKCγ^+/+^ cortical neurons with the same treatment.

To determine the cause of UCHL1 down‐regulation by cPKCγ, we observed the change in protein degradation and mRNA expression of UCHL1 in OGD‐treated neurons. Our results showed that the UCHL1 protein expression was not altered by MG‐132, a proteasome inhibitor, in cPKCγ^−/−^ neurons following 1‐hr OGD/24‐hr R treatment (Fig. 4A and B, *P* > 0.05, *n* = 6 per group). However, the UCHL1 mRNA expression significantly decreased in 1‐hr OGD/24‐hr R‐treated cortical neurons and enhanced in response to cPKCγ gene knockout using agarose gel electrophoresis and real‐time quantitative RT‐PCR (Fig. 4C and D, *P* < 0.001, *n* = 6 per group). Our results above indicate that cPKCγ may decrease mRNA level of UCHL1 rather than its protein degradation.

It has been reported that the functional nuclear factor κB (NF‐κB) response element is found in the UCHL1 promoter region, and NF‐κB activation can suppress UCHL1 gene transcription 35, 36. NF‐κB is activated through the phosphorylation of IκB‐α. Our results showed that the phosphorylation of IκB‐α on Ser‐32 significantly increased after 1‐hr OGD/24‐hr R, but obviously decreased in response to cPKCγ gene knockout (Fig. 4E and F, *P* < 0.001, *n* = 6 per group). These results above indicate that cPKCγ may inhibit the mRNA level of UCHL1 *via* IκB‐α/NF‐κB pathway.

### UCHL1 could aggravate the cortical neuron injuries induced by 1‐hr OGD/24‐hr R treatment

**Figure 5:**
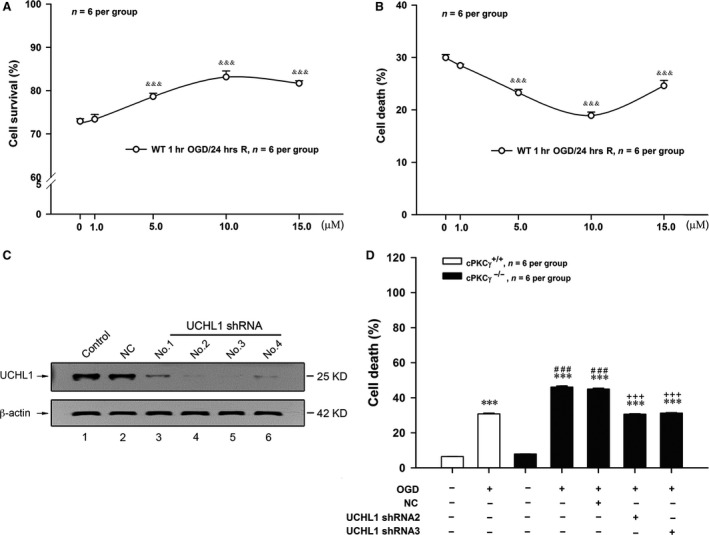
Effects of UCHL1 on the rates of cell survival and death of cultured cortical neurons after 1‐hr OGD/24‐hr R. Quantitative results showed the effects of different doses of LDN on the rate of cell survival (**A**) and the rate of cell death (**B**) induced by OGD treatment (*n* = 6 per group). (**C**) Representative results of Western blot showed UCHL1 shRNA2 and shRNA3 could obviously decrease UCHL1 protein expression, especially. (**D**) Quantitative analysis showed the effects of UCHL1 shRNA2 and shRNA3 treatments on the rate of cell death from Normoxia and OGD groups of cPKCγ^+/+^ and cPKCγ^−/−^ cortical neurons (*n* = 6 per group), respectively. ^&&&^ *P <* 0.001 *versus* vehicle of LDN group with the same treatment; ***P < 0.001 *versus* Normoxia of cPKCγ^+/+^ neurons; ^###^ *P <* 0.001 *versus* corresponding cPKCγ^+/+^ cortical neurons with the same treatment; ^+++^ *P <* 0.001 *versus* NC with the same treatment.

To figure out the possible role of UCHL1 in OGD‐induced ischaemic injuries, LDN, the UCHL1 inhibitor, was used in four different dosages of 1.0, 5.0, 10.0 and 15.0 μM. As shown in Figure 5A and B, LDN (5, 10 and 15 μM) significantly increased the cell survival rate and simultaneously decreased the cell death rate in the cortical neurons with 1‐hr OGD/24‐hr R treatment (Fig. 5A and B, *P* < 0.001, *n* = 6 per group). These results illustrate that UCHL1 may aggravate 1‐hr OGD/24‐hr R‐induced cortical neuron injuries. Consequently, 10 μM LDN was chosen in the following experiments.

To explore whether cPKCγ alleviated ischaemic injury in cultured cortical neurons through inhibiting UCHL1 expression, four UCHL1 shRNA candidates were used. UCHL1 shRNA2 and 3 which more effectively inhibited UCHL1 expression (Fig. 5C) were chosen in the following experiments. As shown in Figure 5D, UCHL1 shRNA 2 and 3 treatments reversed the increased cell death rate induced by cPKCγ gene knockout in 1‐hr OGD/24‐hr R‐treated neurons (*P* < 0.001, *n* = 6 per group).

### Down‐regulation of UCHL1 mediated by cPKCγ protected neurons against ischaemic stroke through autophagy

**Figure 6:**
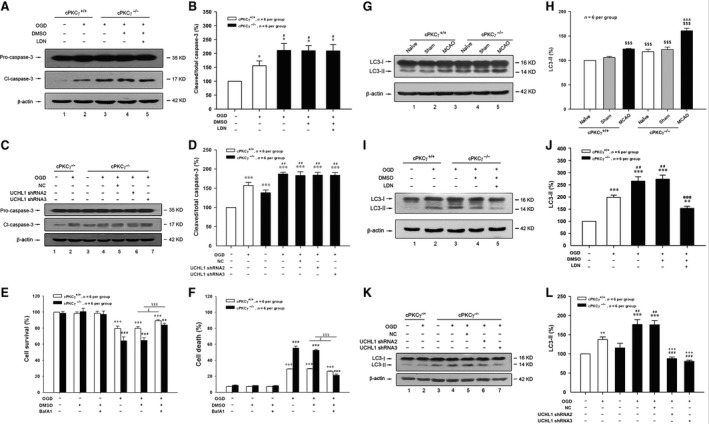
Effects of UCHL1 on the expression of caspase‐3 or LC3 in cortical neurons after 1‐hr MCAO/24‐hr R or 1‐hr OGD/24‐hr R. Representative results (**A** and **C**) and quantitative analysis (**B** and **D**) of Western blot showed the effects of LDN (10.0 μM) and UCHL1 shRNA treatments on the expressions of Pro‐caspase‐3 and Cl‐caspase‐3 from Normoxia and OGD groups of cPKCγ^+/+^ and cPKCγ^−/−^ cortical neurons (*n* = 6 per group), respectively. Quantitative results showed the effects of autophagy inhibitor BafA1 (100.0 nM) on the rate of cell survival (**E**) and the rate of cell death (**F**) from Normoxia and OGD groups of cPKCγ^+/+^ and cPKCγ^−/−^ cortical neurons (*n* = 6 per group), respectively. Representative result (**G**) and quantitative analysis (**H**) of Western blot showed the expression of LC3‐I and LC3‐II in the cortex from Naïve, Sham and MCAO groups of cPKCγ^+/+^ and cPKCγ^−/−^ mice (*n* = 6 per group), respectively. Representative results (**I** and **K**) and quantitative analysis (**J** and **L**) of Western blot showed the effects of LDN (10.0 μM) and UCHL1 shRNA treatments on the expressions of LC3‐II and LC3‐I from Normoxia and OGD groups of cPKCγ^+/+^ and cPKCγ^−/−^ cortical neurons (*n* = 6 per group), respectively. ***P < 0.001, **P < 0.01, *P < 0.05 *versus* Normoxia of cPKCγ^+/+^ neurons; ^###^ *P <* 0.001, ^##^ *P <* 0.01, ^#^ *P <* 0.05 *versus* corresponding cPKCγ^+/+^ cortical neurons with the same treatment; ^£^ *P <* 0.05 *versus* OGD of cPKCγ^+/+^ neurons without BafA1 treatment; ^¥¥¥^ *P <* 0.001 *versus* OGD of cPKCγ^−/−^ neurons without BafA1 treatment; ^$$$^ *P <* 0.001 *versus* NaÏve of cPKCγ^+/+^ mice; ^&&&^ *P <* 0.001 *versus* corresponding cPKCγ^+/+^ mice with the same treatment; ^@@@^ *P <* 0.001 *versus* vehicle of LDN group with the same treatment; ^+++^ *P <* 0.001 *versus* NC with the same treatment.

**Figure 7:**
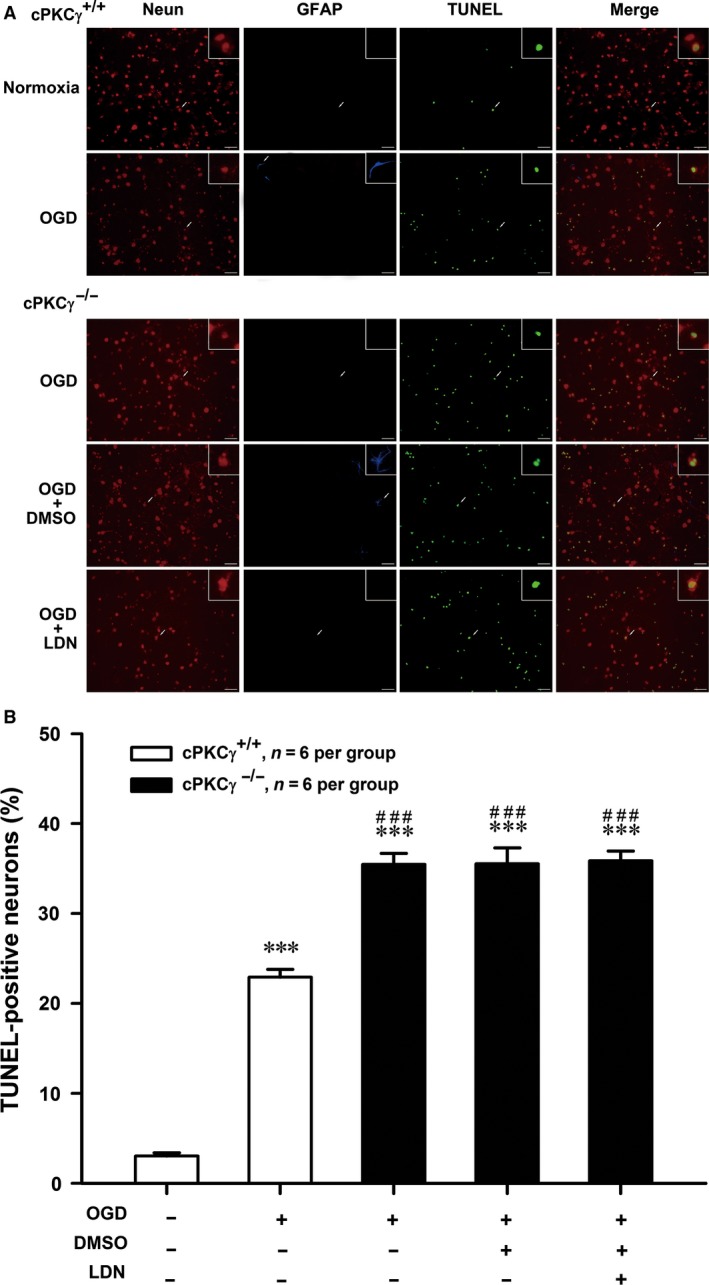
Effects of UCHL1 on the level of TUNEL‐positive neurons in cortical neurons after 1‐hr OGD/24‐hr R. Merged images (**A**) of NeuN (*red*), GFAP (*blue*) and TUNEL (*green*) and quantitative analysis (**B**) showed the effects of LDN (10.0 μM) on the level of TUNEL‐positive neurons from Normoxia and OGD groups of cPKCγ^+/+^ and cPKCγ^−/−^ cortical neurons (*n* = 6 per group), respectively. ***P < 0.001 *versus* Normoxia of cPKCγ^+/+^ neurons; ^###^ *P <* 0.001 *versus* corresponding cPKCγ^+/+^ cortical neurons with the same treatment. Scale bar = 100 μm.

Apoptosis and autophagy were two major morphologically distinctive forms of programmed cell death 25, 37. To explore the role of cPKCγ and UCHL1 in apoptosis and autophagy induced by MCAO or OGD treatment, the ratios of cleaved/total caspase‐3, the TUNEL staining assay and LC3‐II/total LC3 were detected. We found that the ratio of cleaved/total caspase‐3 significantly increased after 1‐hr OGD/24‐hr R and further increased in cPKCγ^−/−^ neuron compared with that of cPKCγ^+/+^ neuron (Fig. 6A and B, *P* < 0.05, *n* = 6 per group; Fig. 6C and D, *P* < 0.01, *n* = 6 per group), but was not altered by LDN or UCHL1 shRNA treatment (Fig. 6A‐D, *P* > 0.05, *n* = 6 per group). The TUNEL staining assay was conducted to assess potential apoptosis; NeuN and glial fibrillary acidic protein (GFAP) were used to identify neurons and astrocytes, respectively. Consistent with the results of caspase‐3, the 1‐hr OGD/24‐hr R treatment also led to a significant increase of the TUNEL‐positive neurons in cPKCγ^−/−^ mice compared with that of cPKCγ^+/+^ mice, but was not altered by LDN treatment (Fig. 7A and B, *P* < 0.001, *n* = 6 per group). These results suggest that UCHL1 does not involve in the effect of cPKCγ on neuronal apoptosis.

Moreover, the conversion of LC3‐I to LC3‐II evidently increased after 1‐hr OGD/24‐hr R or 1‐hr MCAO/24‐hr R treatments (Fig. 6G and H, *P* < 0.001, *n* = 6 per group; Fig. 6I and J, *P* < 0.001, *n* = 6 per group; Fig. 6K and L, *P* < 0.01, *n* = 6 per group). The role of autophagy in ischaemic stroke is still controversial. Therefore, BafA1, an autophagy inhibitor, was used to determine the role of autophagy in the ischaemic neuronal injury. BafA1 treatment was found to increase the cell survival rate and simultaneously decrease the cell death rate in both cPKCγ^+/+^ and cPKCγ^−/−^ neurons after 1‐hr OGD/24‐hr R (Fig. 6 E and F, *P* < 0.05 or 0.001, *n* = 6 per group), which indicate that autophagy may aggravate ischaemic neuronal injuries. The high ratio of LC3‐II/total LC3 induced by MCAO or OGD treatment was further increased by cPKCγ gene knockout and then was significantly decreased by LDN or UCHL1 shRNA treatment (Fig. 6G and H, *P* < 0.001, *n* = 6 per group; Fig. 6I‐L, *P* < 0.01, *n* = 6 per group). Our results demonstrate that down‐regulation of UCHL1 might involve in the effect of cPKCγ on autophagy during ischaemic injury.

### Down‐regulation of UCHL1 mediated by cPKCγ decreased OGD‐induced autophagy through ERK‐mTOR pathway

**Figure 8:**
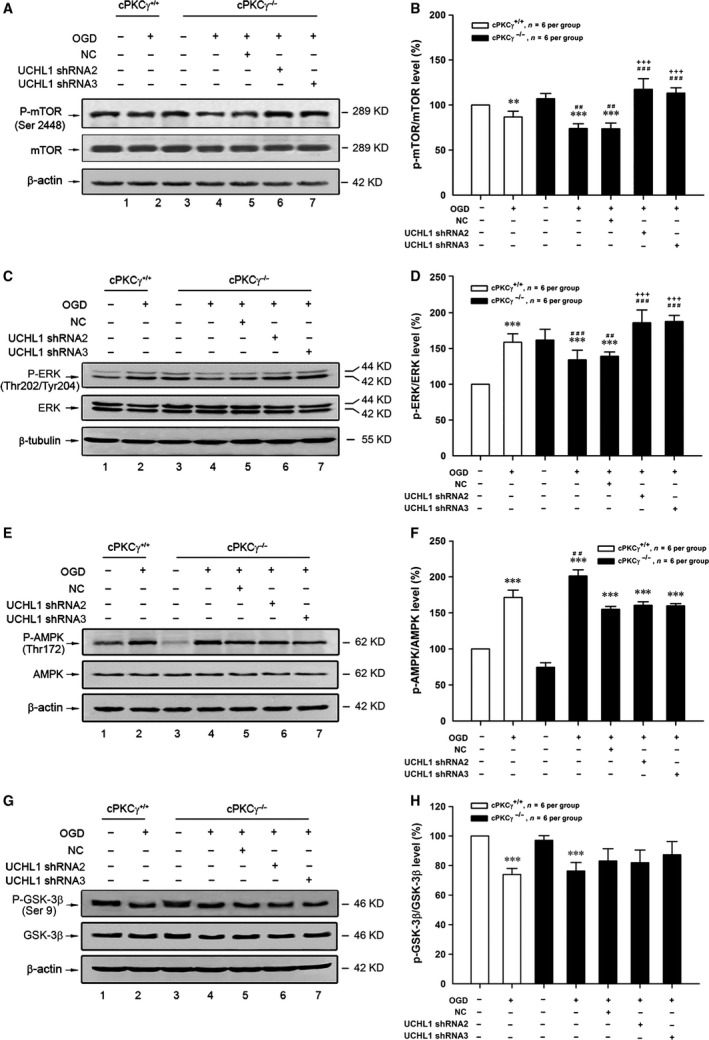
Effects of UCHL1 on the expression of mTOR, ERK, AMPK and GSK‐3β in cortical neurons after 1‐hr OGD/24‐hr R. Representative results of (**A**,** C**,** E** and **G**) and quantitative analysis (**B**,** D**,** F** and **H**) of Western blot showed the effects of UCHL1 shRNA treatments on the phosphorylation levels of mTOR, ERK, AMPK, GSK‐3β from Normoxia and OGD groups of cPKCγ^+/+^ and cPKCγ^−/−^ cortical neurons (*n* = 6 per group), respectively. ***P < 0.001, **P < 0.01 *versus* Normoxia of cPKCγ^+/+^ neurons; ^###^ *P <* 0.001, ^##^ *P <* 0.01 *versus* corresponding cPKCγ^+/+^ cortical neurons with the same treatment; ^+++^ *P <* 0.001 *versus* NC with the same treatment.

To explore the roles of cPKCγ and UCHL1 in OGD‐induced autophagy *in vitro*, the autophagy‐related protein expressions were observed. Our results showed that 1‐hr OGD/24‐hr R treatment could obviously reduce mTOR phosphorylation (P‐mTOR) and cPKCγ gene knockout resulted in more reduction in P‐mTOR (Fig. 8A and B, *P* < 0.01, *n* = 6 per group). However, UCHL1 shRNA 2 and 3 treatments could significantly enhance the levels of P‐mTOR in cPKCγ^−/−^ neurons with OGD treatment (Fig. 8A and B, *P* < 0.001, *n* = 6 per group).

It is generally known that ERK, AMPK and GSK‐3β can regulate the phosphorylation of mTOR 38, 39, 40. We found the phosphorylation of ERK (P‐ERK) significantly decreased in the cPKCγ^−/−^ neurons compared with cPKCγ^+/+^ neurons after 1‐hr OGD/24‐hr R treatment (Fig. 8C and D, *P* < 0.001, *n* = 6 per group). UCHL1 shRNA 2 and 3 treatments could significantly enhance the level of P‐ERK (Fig. 8C and D, *P* < 0.001, *n* = 6 per group), which was consistent with the change of P‐mTOR.

In addition, the phosphorylation level of AMPK obviously increased after 1‐hr OGD/24‐hr R and further increased in the cPKCγ^−/−^ neurons compared with that of cPKCγ^+/+^ neurons (Fig. 8E and F, *P* < 0.01, *n* = 6 per group), but was not altered by UCHL1 shRNA 2 and 3 treatments (Fig. 8E and F, *P* > 0.05, *n* = 6 per group). At the same time, the phosphorylation level of GSK‐3β obviously decreased after 1‐hr OGD/24‐hr R (Fig. 8G and H, *P* < 0.001, *n* = 6 per group), but was altered by neither cPKCγ gene knockout nor UCHL1 shRNAs (Fig. 8G and H, *P* > 0.05, *n* = 6 per group). These results indicate that down‐regulation of UCHL1 mediated by cPKCγ may decrease OGD‐induced autophagy *via* ERK‐mTOR pathway.

## Discussion

cPKCγ is ubiquitously expressed in the central nervous system 41, which results in much attention in exploring its roles in brain injury. In this study, we found cPKCγ knockout could aggravate the neurological deficits which could be proved by neurological score and behavioural tests and increase the infarct volume of mice with ischaemic stroke. Simultaneously, the experiments *in vitro* showed that lack of cPKCγ could decrease the survival of OGD‐treated primary cortical neurons as well. Our results further validate that endogenous cPKCγ protein may be beneficial to neuronal survival and improve stroke outcome.

The localization of UCHL1 is also restricted to neurons. The significant elevation of UCHL1 in CSF and serum is found in the traumatic brain injury (TBI) patients and rats subjected to MCAO, which may be correlated with injury severity and survival outcome of brain injuries 22, 23, 42. In this study, we observed the same change in CSF and serum of mice with 1‐hr MCAO/24‐hr R treatment. Because UCHL1 is a neuronal‐specific protein, the elevated UCHL1 levels in CSF and serum should be due to its release by neurons. Therefore, we detected the level of UCHL1 in culture medium of primary mouse cortical neurons. The results showed that the level of UCHL1 obviously increased in culture medium following 1‐hr OGD/24‐hr R. We also observed the protein expression of UCHL1 in neurons. Our results showed that both 1‐hr MCAO/24‐hr R and 1‐hr OGD/24‐hr R treatments could obviously decrease the protein expression of UCHL1. The UCHL1 inhibitor and shRNAs significantly increased the cell survival of cortical neurons with 1‐hr OGD/24‐hr R treatment, indicating that UCHL1 may aggravate 1‐hr OGD/24‐hr R‐induced cortical neuron injuries. Furthermore, we found that cPKCγ knockout did not affect the release of UCHL1 by neurons, while increased the protein expression of UCHL1 in cortical neurons after MCAO or OGD, indicating that down‐regulation of UCHL1 mediated by cPKCγ may protect neurons against ischaemic injuries. We next tested whether the increase in UCHL1 expression was determined by its protein degradation or mRNA level. We found that the proteasome inhibitor did not alter the UCHL1 protein expression of cPKCγ^−/−^ neurons following 1‐hr OGD/24‐hr R. However, the UCHL1 mRNA expression of cortical neurons significantly decreased after 1‐hr OGD/24‐hr R and increased in response to cPKCγ gene knockout, which was similar to that of the UCHL1 protein expression. Our results indicate that cPKCγ may decrease UCHL1 protein expression *via* lowering the mRNA level rather than its protein degradation.

Recently, a functional NF‐κB response element is identified in the UCHL1 promoter region, and it has been reported that NF‐κB activation suppressed UCHL1 gene transcription 35, 36. NF‐κB complex mainly includes the p65, p50 and NF‐κB inhibitor alpha (IκB‐α) subunit 43. The IκB‐α is the most frequent expressed form of NF‐κB inhibitors in the nervous system 44. Various phosphorylation sites (S32, S36 and Y42) within IκB‐α have been demonstrated to be important in regulating NF‐κB activation 45. NF‐κB is activated through the phosphorylation of IκB‐α which promotes to release NF‐κB into nuclear and binds to the promoter sites of target genes. Several studies have shown that PKCθ, PKCζ, PKCδ and aPKC are critical for activation of NF‐κB in T cell, monocyte, macrophage, epithelial cell, endothelial cell and microglia, respectively 46, 47, 48, 49, 50, 51. The PKC inhibitor can inhibit the phosphorylation of IκB‐α and decelerate the degradation of IκB‐α 52. In this study, we observed that the phosphorylation of IκB‐α (Ser 32) significantly increased after OGD but decreased in response to cPKCγ gene knockout. Our results suggest that cPKCγ may down‐regulate UCHL1 mRNA expression through the IκB‐α/NF‐κB pathway.

It is generally known that oxidative stress and apoptosis are involved in brain damage after cerebral ischaemia 25, 53. In this study, we found that cPKCγ gene knockout significantly enhanced OGD‐induced oxidative stress (data not shown) and apoptosis, indicating that cPKCγ may exert neuroprotective effects against oxidative stress and apoptosis during ischaemic injury. UCHL1 is found to increase cellular ROS level by up‐regulating H_2_O_2_ generation in melanoma cells and induce apoptosis in spermatocyte of mice with cryptorchidism and breast tumour cells 54, 55, 56. Then, we explored whether cPKCγ could regulate oxidative stress and apoptosis through UCHL1 in the present study. We found that UCHL1 inhibitor or various shRNAs failed to affect oxidative stress and apoptosis induced by OGD treatment in cPKCγ^−/−^ neurons. Our results suggest that UCHL1 may not be involved in the effects of cPKCγ on oxidative stress and apoptosis during ischaemic injury.

In recent years, more attention has been paid to the role of autophagy in ischaemic injuries. However, the role of autophagy in ischaemic stroke is still controversial. Induction of autophagy by nicotinamide phosphoribosyltransferase increases the cell survival rate and simultaneously decreases the LDH leakage of primary neurons with 2‐hr OGD treatment 57. But other studies have reported that activation of autophagy can aggravate cortical neural cell injury following 1‐hr focal cerebral ischaemia/24‐hr R 58, 59. Our results showed that the ratio of LC3‐II/total LC3, related with the level of autophagosome formation, evidently increased after 1‐hr MCAO/24‐hr R or 1‐hr OGD/24‐hr R treatment. BafA1, an autophagy inhibitor, could increase the cell survival rate and simultaneously decrease the cell death rate of cortical neurons with 1‐hr OGD/24‐hr R treatment, suggesting autophagy may be involved in a neuronal death pathway following ischaemic neuronal injury. In addition, we found that cPKCγ knockout could significantly increase the conversion of LC3‐I to LC3‐II in peri‐infarct region of mice with ischaemic stroke and primary cultured cortical neurons with OGD treatment, suggesting cPKCγ can negatively regulate autophagy induced by ischaemic neuronal injury. Inhibition of UCHL1 activity by LDN treatment is reported to regulate activation of the autophagy pathway in α‐syn tg mice and α‐syn over expressing cells 60. Then, we explored whether cPKCγ could regulate autophagy through UCHL1. We observed that both LDN and UCHL1 shRNA treatments significantly decreased the ratio of LC3‐II/total LC3‐I in cPKCγ^−/−^ neurons, suggesting that UCHL1 is involved in the effect of cPKCγ on autophagy following ischaemic neuronal injury.

Autophagy is a bulk protein degradation system that is involved in multiple cellular processes. The kinase mTOR is a considerable regulator of autophagy induction, when regulating mTOR positively can suppress autophagy and inhibiting mTOR can promote it. Rapamycin which directly inhibits mTOR is the most commonly used and specific inducer of autophagy 61. Our results showed that the mTOR phosphorylation level obviously decreased after 1‐hr OGD/24‐hr R, what's more, cPKCγ knockout resulted in more reduction in P‐mTOR. However, UCHL1 shRNAs could significantly enhance the mTOR phosphorylation in the cPKCγ^−/−^ neurons with OGD treatment. ERK, one of the upstream targets of mTOR, can positively regulate mTOR activation and inhibit autophagy flux 62. The mTOR can be inhibited by AMPK which regulates intracellular energy status by sensing the AMP/ATP ratio 63. GSK‐3β is also generally known to be a negative regulator of mTOR, when inactivating GSK‐3β by phosphorylation leads to the activation of mTOR signalling pathways 64. The inhibition of PKC by GF‐109203X can activate GSK‐3, providing evidence that the activity of GSK‐3 is negatively regulated by PKC 24. Our results showed that consistent with the trend of P‐mTOR, cPKCγ knockout significantly decreased the ERK phosphorylation following 1‐hr OGD/24‐hr R, while UCHL1 shRNAs obviously enhanced the level of P‐ERK. The phosphorylation of AMPK significantly increased after 1‐hr OGD/24‐hr R, further increased in the cPKCγ^−/−^ neurons, but was not altered by various UCHL1 shRNA treatments. The phosphorylation of GSK‐3β obviously reduced after 1‐hr OGD/24‐hr R, but was neither altered by cPKCγ knockout nor UCHL1 shRNAs. These results above suggest that cPKCγ/UCHL1 may regulate autophagy through ERK‐mTOR pathway during ischaemic neuronal injury.

## Conclusion

**Figure 9:**
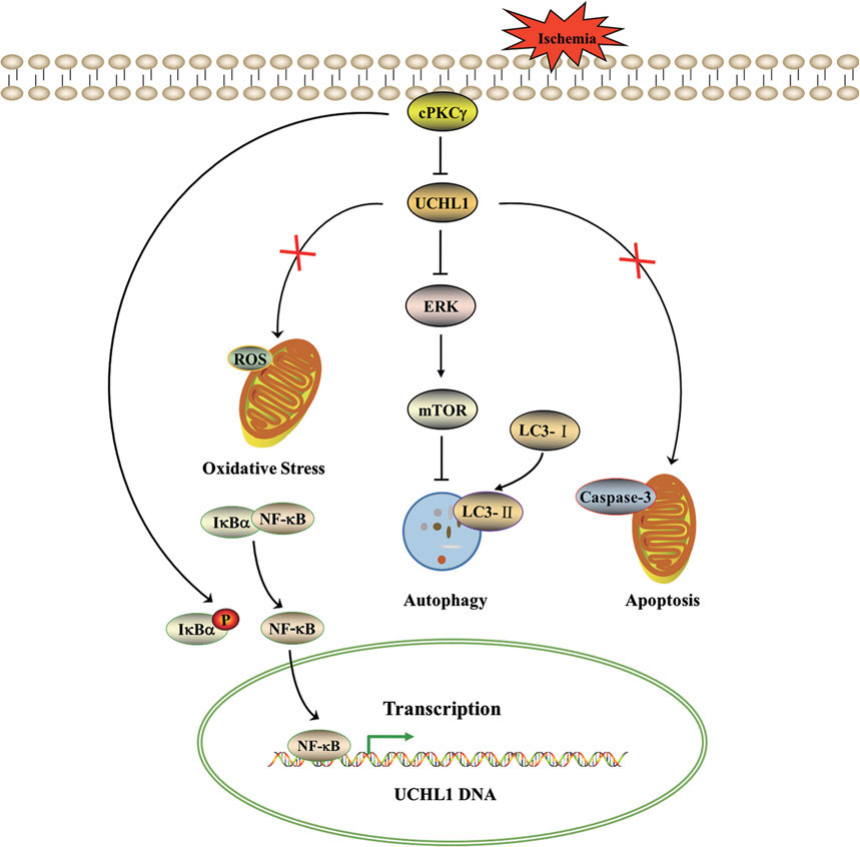
Schematic diagram of the effect of cPKCγ‐UCHL1 on autophagy through ERK‐mTOR pathway in cerebral ischaemic injuries. cPKCγ reduces UCHL1 protein expression *via* decreasing its mRNA level regulated by the IκB‐α/NF‐κB pathway in cortical neurons following ischaemic neuronal injuries. UCHL1 positively regulates autophagy through ERK‐mTOR pathway, which may aggravate cerebral ischaemic injuries, instead of oxidative stress and apoptosis.

In summary, our results indicate that down‐regulation of UCHL1 mediated by cPKCγ may negatively regulate autophagy through ERK‐mTOR pathway, which alleviates ischaemic neuronal injury (Fig. 9). However, further studies about the mechanism how UCHL1 can regulate ERK‐mTOR pathway will be required.

## Conflict of interest

The authors confirm that there are no conflict of interests.
